# Changes in Corneal Parameters after DMEK Surgery: A Swept-Source Imaging Analysis at 12-Month Follow-Up Time

**DOI:** 10.1155/2021/3055722

**Published:** 2021-07-21

**Authors:** Anna Machalińska, Agnieszka Kuligowska, Karolina Kaleta, Monika Kuśmierz-Wojtasik, Krzysztof Safranow

**Affiliations:** ^1^First Department of Ophthalmology, Pomeranian Medical University, Szczecin, Poland; ^2^Faculty of Graphics, Academy of Art, Szczecin, Poland; ^3^Department of Biochemistry and Medical Chemistry, Pomeranian Medical University, Szczecin, Poland

## Abstract

**Purpose:**

To assess the time course changes in corneal topographic parameters during the one-year follow-up after Descemet membrane endothelial keratoplasty (DMEK) surgery.

**Materials and Methods:**

Twenty-one patients (24 eyes) who underwent DMEK surgery were evaluated. Best corrected visual acuity (BCVA), endothelial cell count (ECC), central corneal thickness (CCT), mean keratometry (MK), mean astigmatism (MA), astigmatism asymmetry (AA), and higher-order aberration (HOA) were assessed at baseline and 1, 3, 6 and 12 months after the surgery using CASIA2 anterior segment swept-source OCT (Tomey, Japan).

**Results:**

In patients who underwent DMEK surgery, BCVA improved gradually at the subsequent visits during the 12-month follow-up. A significant reduction in ECC and CCT at the 1st month was noted, which remained stable until the 6th month postoperatively. Anterior and total MK values remained unchanged, whereas changes in posterior keratometry were noticeable until the 6th month after surgery. A significant reduction in the anterior, posterior, and total astigmatism magnitude as well as astigmatism asymmetry was observed during the first 6 months after surgery. A gradual anterior, posterior, and total HOA decrease was documented until the 12th month after surgery. Negative correlations between baseline values of CCT, MK, MA, AA, and HOA and postoperative variations in those parameters at consecutive follow-up time points were observed. Accordingly, negative correlations between baseline CCT and postoperative changes in corneal topographic parameters after surgery were found.

**Conclusion:**

The stabilization of most corneal topographic parameters takes place within 6 months after the procedure, whereas HOA reduction and BCVA improvement gradually occur during the first year after surgery. Preoperative values of corneal topographic parameters strongly determine their changes detected after DMEK surgery, which may suggest that early therapeutic intervention results in better visual outcomes.

## 1. Introduction

In the last few years, Fuchs corneal endothelial dystrophy and pseudophakic bullous keratopathy have become the most common indications for corneal transplantation [1]. Less than 25 years ago, the introduction of endothelial keratoplasty (EK) by Melles in 1998 revolutionized corneal transplantation [2] and was a salvation for patients with corneal endothelial disease. The introduction of the Descemet membrane endothelial keratoplasty (DMEK) technique, a selective replacement of the Descemet membrane and its endothelium, has resulted in significant progress in lamellar corneal surgery [3, 4]. Following the first procedures performed in 2006, the popularity of DMEK surgery quickly began to grow. DMEK, next to the other lamellar keratoplasty techniques as Descemet stripping endothelial keratoplasty (DSEK) or Descemet stripping automated endothelial keratoplasty (DSAEK), began to replace the conventional penetrating keratoplasty (PKP) for selective replacement of the diseased posterior layers of the cornea in patients with endothelial insufficiency [5]. Furthermore, it soon became evident that the near complete restoration of the corneal anatomy with the DMEK technique provided unprecedented visual outcomes and an even lower risk of allograft rejection [5–8]. Moreover, there was no need for expensive and specialized equipment, such as a microkeratome or femtosecond laser, for the preparation of the donor tissue while conducting DMEK surgery [9].

Since the introduction of the DMEK technique, changes in endothelial cell count (ECC), best corrected visual acuity (BCVA), and central corneal thickness (CCT) after surgery have been widely studied [7, 10–13]. There are only a few reports analyzing postoperative corneal aberrations [14–16], keratometry [17–19], and astigmatism changes [20–22]. Regrettably, there are few studies that assess the dynamics of changes in corneal parameters over time in a detailed way.

In the present study, we aimed to assess the time course changes in corneal topographic parameters during a one-year follow-up after DMEK surgery. We also explored the relationship between dynamic variations in corneal curvature, CCT, BCVA, and ECC between individual visits throughout the 12-month postoperative observation time. To the best of our knowledge, this might be the first retrospective study to provide such a broad and precise analysis of dynamic variations in corneal keratometry, astigmatism, astigmatism asymmetry, and HOA values based on anterior segment swept-source optical coherence tomography (SS-OCT) recordings.

## 2. Materials and Methods

This retrospective case series included 24 eyes after DMEK surgery, which was carried out due to various causes of endothelial decompensation: Fuchs endothelial corneal dystrophy (FECD) or pseudophakic bullous keratopathy (PBK). The patients enrolled in the study were operated on at the First Ophthalmology Clinic in Szczecin in 2018–2020 and then monitored 1, 3, 6, and 12 months after the surgery. All participants underwent a complete ophthalmologic examination, including the following: best corrected distance visual acuity with Snellen charts, slit lamp biomicroscopy, and a detailed fundus examination after pupil mydriasis. Intraocular pressure (IOP) measurement and corneal quality parameters were evaluated with swept-source anterior segment optical coherence tomography (AS-OCT) at each follow-up visit.

### 2.1. Surgical Technique and Graft Preparation

Donor corneas were obtained with the multiorgan procurement method and in the dissecting room during autopsy. Corneoscleral buttons were stored in Eusol-C medium (Alchimia, Italy) in hypothermic storage at 2–6°C at the West Pomeranian Eye Tissue Bank in Szczecin. The prestorage evaluation of the endothelium was performed by specular microscopy (Konan CellCheck EB-10, Konan Medical, USA). All corneas had an endothelial cell count of at least 2800 cells/mm^2^.

Direct preparation of the graft before transplantation took place in the operating theatre. All grafts were stripped and left on their natural support immersed in 0.06% trypan blue dye (Vision blue; D.O.R.C). Then, grafts were trephined by the surgeon to the desired diameter (6.0–8.0 mm) using a Hessburg-Barron donor corneal punch (Barron Precision Instruments, USA).

Each surgery was performed by the same surgeon. All patients underwent prophylactic basal laser iridectomy at 6 o'clock position before endothelial keratoplasty to minimize the risk of postoperative pupillary block. Patients with retinal diseases significantly affecting visual acuity were excluded from the study. All procedures were performed with peribulbar block.

The epithelium of the recipient was marked to guide the subsequent Descemetorhexis and to allow the correct positioning and perfect centring of the transplanted donor flap. The anterior chamber (AC) of the eye was then entered through a clear corneal incision. After the injection of a hyaluronate viscoelastic material, the endothelium and the Descemet membrane were stripped using an inverted Price-Sinskey hook. The stripping diameter was 1 mm wider than the graft diameter. The viscoelastic material was rinsed after stripping. The removed flap was exposed on the anterior surface of the receiver's cornea to verify its integrity.

The surgery was performed following the “no-touch” technique. The trephined DMEK graft was carefully detached from the surrounding Descemet membrane, immersed in sterile balanced salt solution, and aspirated into the transparent glass cartridge (Geuder AG, Germany). The rolled graft was injected into the AC with slow and continuous pressure through the main incision (2.4 mm). The graft was then unfolded and positioned using the tap-tap technique. After ensuring the correct orientation and centration, the graft was pressed against the recipient stroma by injecting SF6 underneath. One nonabsorbable 10-0 suture was applied to the operating port and kept until the first-week follow-up visit.

All patients were instructed to stay in a supine position until the first postoperative flap position control was done. In the case of a pupillary block or ocular hypertension, topical mydriatics were administered, or if this procedure was insufficient, a small quantity of air was released from the AC in the operating theatre. The postoperative treatment consisted of a topical antibiotic given 4 times a day for 1 week and topical preservative-free dexamethasone sodium phosphate 8 times a day for the first month. The topical steroid was tapered down to one drop every other day and then discontinued over a 1-year period.

### 2.2. Visual Acuity and Endothelial Cell Count Measurements

Visual function was assessed in all participants by evaluating BCVA using a Snellen chart. The result was recorded in the decimal system.

The ECC was measured at each follow-up visit using a specular microscope (EM-4000, Tomey, Japan).

### 2.3. AS-OCT Measurements

Both corneal thickness and keratometry values were determined using a swept-source anterior segment OCT CASIA2 (Tomey, Japan). During the entire observation period, the CASIA2 was placed in the same room under the same lighting conditions. All measurements were taken by trained operators. Operators gently held patients' eyelids to avoid putting pressure to the globe. The scan was performed using the autoalignment function. The CASIA 2 measurements were obtained with the corneal map mode of the anterior segment module. The images were analyzed by built-in 2D analysis software that automatically calculated the measurements along with the structural outlines and reference lines. The outline tracer was edited where needed.

Central corneal thickness (CCT) (*μ*m), mean keratometry values (D), astigmatism power (D) and axis (°), astigmatism asymmetry (D), and higher-order aberration (HOA) power (D) were recorded and analyzed at assumed time points after surgery using the Fourier analysis 3D/2D function. Measurements were read from both the anterior and posterior surfaces of the cornea, and the total values were taken into account. All parameters were assessed at optical zones (OZs) with 3 and 6 mm diameter. The image quality was assessed during acquisition by the operator. Only well centred measurements with high-quality indexes were included in the study.

### 2.4. Statistical Analysis

The statistical analysis was conducted using Statistica software. Parametric variables were established by the Shapiro–Wilk test. The Wilcoxon signed-rank test was used to compare the preoperative and postoperative values. The correlations between the baseline variables and the corneal parameters were analyzed with Spearman's rank correlation coefficient (Rs). A *p* value of less than 0.05 was considered significant.

## 3. Results

### 3.1. Baseline Characteristics of the Study and Donor Groups

Twenty-four eyes of 21 patients qualified for DMEK surgery (*n* = 24). No graft failures or rejections were observed. The preoperative characteristics of the patients, as well as donor graft characteristics, are shown in Table 1. The study group consisted of 5 men and 16 women. The mean age of the patients was 66.25 ± 11.23 years. All men and 12 women underwent surgery secondary to FECD. In 4 women, the indication for surgery was PBK. At baseline, the mean values of BCVA and CCT were 0.2 and 680.50 *μ*m, respectively.

### 3.2. The Influence of DMEK Surgery on BCVA

**Figure 1:**
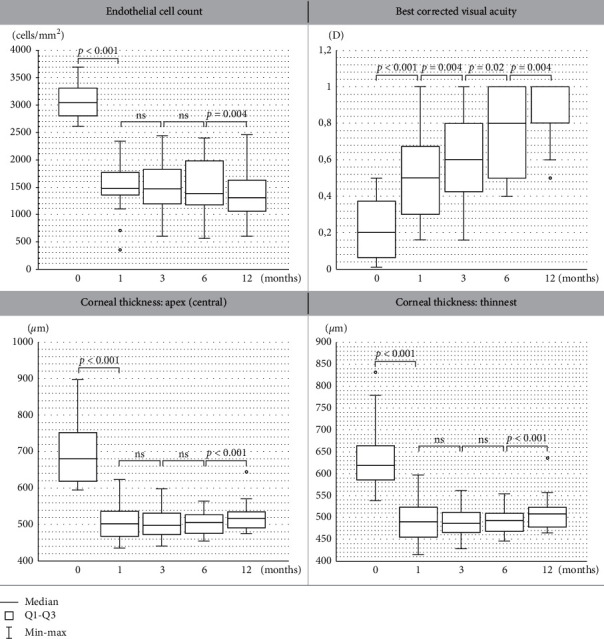
Boxplots showing (a) endothelial cell count (ECC), (b) best corrected visual acuity (BCVA), and corneal thickness ((c) apex-central and (d) thinnest) at baseline and at the 1st, 3rd, 6th, and 12th month after DMEK surgery. ns = not significant.

First, the influence of DMEK surgery on BCVA improvement was evaluated (Figure 1(a)). The median baseline BCVA was 0.2, and it increased gradually at the consecutive follow-up visits (median = 0.5 at the 1st month, median = 0.6 at the 3rd month, median = 0.8 at the 6th month, and median = 1 at the 12th month).

### 3.3. The Dynamics of Changes in Endothelial Cell Count and Central Corneal Thickness

Next, the dynamics of the changes in endothelial cell count after DMEK surgery were analyzed (Figure 1(b)). A significant decrease of 51,67% in ECC value was noted after 1st month compared to baseline (median = 3045.5 cells/mm^2^ at baseline and median = 1472 cells/mm^2^ at the 1st month after surgery; *p* < 0.001). No significant changes in ECC values (cells/mm^2^) at the 1st, 3rd, and 6th month after surgery were noted. Subsequently, we observed a significant reduction in ECC at the 12th month compared to the values recorded at the 6th month.

Regarding corneal thickness, in comparison with the baseline values (*μ*m) (Figure 1(c)), CCT decreased significantly at the 1st month after the procedure, remaining stable and unchanged from the 1st month until the 6th month postoperatively. Then, we observed a significant increase in CCT at the 12th month compared to the values recorded at the 6th month.

### 3.4. The Dynamics of Changes in Corneal Topography

Further, we analyzed the dynamics of changes in keratometry recordings after DMEK surgery (Table 2). No significant changes in the magnitude of anterior keratometry values (D) between the follow-up time points were noted. Similarly, we observed no significant changes in total keratometry between time points with only one exception: the values noted at the 1st month postoperatively were lower than those recorded preoperatively exclusively in the 6 mm OZ group. This could suggest that the DMEK procedure might not have an impact on anterior and total keratometry values. Regarding the posterior corneal surface, we observed a significant reduction in keratometry values at the 1st month compared to baseline values. Then, a significant increase in posterior keratometry recordings at the 3rd month compared to the 1st month was noted. Accordingly, the values obtained at the 6th month were higher than those observed at the 3rd month postoperatively. The posterior keratometry recordings stabilized at the 6th month, remaining unchanged until the 12th month after surgery (median = −6.34 D for 3 mm OZ and median = −6.32 D for 6 mm OZ, *p*=0.38 and *p*=0.26, respectively).

Afterwards, the influence of the DMEK procedure on astigmatism changes was evaluated (Table 2). We observed a significant reduction in the anterior, posterior, and total astigmatism magnitude at the 12th month follow-up visit postoperatively compared to baseline values (D). Interestingly, no changes in total astigmatism power were detected directly after the procedure at the 1st month after DMEK procedure compared to preoperative values. A subsequent reduction in total astigmatism power was detected only at the 3rd month after the procedure compared to values recorded at the 1st month visit, with subsequent stabilization of astigmatism power from the 3rd month up to the 12th month of follow-up. A similar pattern of astigmatism reduction was observed for the anterior and posterior cornea at the 3 mm and 6 mm OZ. Additionally, we noted a significant decrease in posterior astigmatism power at the 6th month compared to baseline values exclusively in the 6 mm OZ. Interestingly, the axis of the baseline total astigmatism remained unchanged throughout the whole follow-up period (data not shown).

Next, we evaluated irregular corneal astigmatism with an asymmetry of the astigmatic components (Table 2). We observed a significant reduction in the anterior, posterior, and total astigmatism asymmetry at the 12th month follow-up visit postoperatively compared to baseline values (D). The follow-up analysis of the astigmatism asymmetry dynamics revealed a significant reduction at the examination conducted 1 month after surgery with subsequent stabilization of this parameter after the 6 months of observation. Accordingly, we found no differences in astigmatism asymmetry (AA) values between the 6-month and 12-month follow-up visits.

To provide a broad-based assessment of corneal topography after DMEK surgery, we analyzed the higher-order corneal aberrations (D) that may influence vision quality and acuity (Table 2). A gradual HOA reduction of the total cornea as well as the anterior HOA after the treatment was observed up to the 12th month after surgery. Regarding posterior HOA, a significant decrease in HOA values was observed at early time points, while the values stabilized at the 6th month and remained unchanged until the 12th month postoperatively. Interestingly, a significant reduction in posterior and total surface HOA was detected directly after the procedure beginning from the 1st month after surgery, while the values of anterior HOA did not decrease until the 3-month follow-up visit.

### 3.5. The Potential Relationships between Corneal Parameters at Consecutive Time Points after Surgery

In the next step, we evaluated the potential relationships between corneal parameters at consecutive time points after surgery. We detected that preoperative values of corneal parameters strongly determined their changes detected after DMEK surgery. Accordingly, we found negative correlations between baseline values of CCT, keratometry, astigmatism, astigmatism asymmetry, and HOA and postoperative variations in those parameters at consecutive follow-up time points (Table 3). This finding indicates that the thicker the cornea and the higher the values of keratometry, astigmatism, astigmatism asymmetry, and HOA preoperatively, the lower the reduction in those parameters postoperatively.

Importantly, we determined that baseline CCT strongly influences the changes in other corneal topographic parameters after surgery.  Table 4 shows the correlations between baseline CCT and the changes in VA, CCT, keratometry, astigmatism, astigmatism asymmetry, and higher-order aberration values after DMEK surgery. We found that preoperative CCT negatively correlated with changes in corneal thickness, astigmatism power, astigmatism asymmetry, and HOA at the following postoperative visits. This result indicates that the thicker the cornea before surgery, the lower the decrease in magnitudes of regular and irregular corneal astigmatism and HOA after DMEK surgery.

## 4. Discussion

Endothelial corneal transplantation techniques are constantly being improved, and their continuous development contributes to more effective and safer treatment of patients with endothelial damage.

Many previous studies have focused on the variations in visual acuity and corneal topographic parameters in 6- or 12-month observation periods, comparing them to the baseline conditions [7, 13, 23, 24]. Very few studies have also analyzed the dynamics of changes in topographic recordings at individual visits during the observation period [19].

This retrospective study provided such detailed analysis of the relationships between the dynamic status of corneal topographic parameters, corneal thickness, best corrected visual acuity, and endothelial cell count analyzed at several postoperative follow-up points throughout a 1-year observation period.

Our data provided evidence that posterior keratometry recordings decreased just after the operation at the 1st month postoperatively compared to those recorded preoperatively, which indicates a steepening of the posterior corneal curvature. Altogether, the potential explanation is that the recipient endothelium is replaced with an undersized donor graft; thus, the peripheral margin of the stripped area is deprived of endothelial cells, causing marginal thickening that is due to the unsealed endothelial cell barrier at the peripheral corneal area. This consequence occurs with subsequent steeping of the posterior corneal surface. With time, the donor cells migrate and fill the gaps between recipient and donor tissue, thus leading to a resolution of the peripheral oedema and a flattening of the posterior corneal surface. Subsequently, the cornea returns to a physiologically hydrated status. Indeed, we found a significant increase in posterior keratometry values at 3 and 6 months after DMEK surgery, with subsequent stabilization at 6 to 12 months. Van Dijk et al. in their large prospective study on DMEK indicated that a potential cause of posterior keratometry decrease is the specific corneal healing process. In the early phase after DMEK surgery, the cornea shows central thinning while the periphery is still edematous, creating a steepening of the posterior cornea curvature and a flattening of the anterior cornea curvature, which results in a “hyperopic shift.” As the transplanted cornea returns to a physiological hydration status, the induced hyperopic shift is again reduced but still detectable in comparison to the preoperative power [6].

In our study posterior keratometry outcomes coincided with a decrease in ECC at the 12-month compared to the 6-month measurements since endothelial cells have limited potential to proliferate. We cannot exclude the possibility that a decrease in ECC results in an increase of CCT at the 12th month postoperatively since we documented an increase in CCT at the 12-month compared to the 6-month values.

Indeed, similar patterns of ECC and CCT changes were documented in previous studies [7, 11, 23–25].

Importantly, Brockmann et al., in their prospective observational study [13], noted that patients with baseline CCT over 625 *μ*m might have a thicker cornea at the 12-month follow-up. This finding is in line with our observation that baseline CCT determines the variations in corneal thickness after surgery since we found a strong negative correlation between baseline and postoperative changes in corneal thickness at subsequent postoperative visits.

Furthermore, we observed that the DMEK procedure did not impact anterior or total keratometry values. This observation is in line with the data of Kwon et al., who documented that total keratometry did not change significantly after the DMEK procedure and that postoperative values were comparable to those in the healthy cohort. Accordingly, the authors found that the anterior corneal surface remained relatively unchanged, whereas the posterior corneal surface displaced forward [19]. On the other hand, Van Dijk et al., in a large prospective study of 217 eyes, evaluated the keratometry outcomes of patients after the DMEK procedure and showed a pre- to postoperative change in the spheric equivalent of +0.41 ± 1.06 D for the whole study group [6]. Similarly, Ham et al., in a study of 50 eyes, showed a pre- to postoperative hyperopic shift of +0.32 ± 1.01 D at the 6-month follow-up after DMEK surgery [26]. A change in total refractive corneal power was the result of posterior surface MK change, since the anterior corneal curvature in Scheimpflug imaging was stable. Nevertheless, the authors concluded that normal intraocular power nomograms for cataract surgery should be applied before or during DMEK surgery. Dirisamer et al. demonstrated nearly the same behavior of pre- to postoperative refractive changes [27]. On the other hand, the data presented by Alnaweiseh et al. show a significant change in the refractive power of the posterior surface of the cornea and thus a decrease in the total refractive power of approximately 1 D, whereas the anterior cornea remained nearly unchanged [18]. Interestingly, the retrospective cohort study published by Fritz et al. has proven that patients with centrally flatter, oblate posterior corneas (positive posterior Q) are at higher risk of having postoperative hyperopic shift than other patients. Authors suggest that subtracting 0.5 D of planned refraction before conducting triple procedure (combined DMEK and cataract surgery) in those eyes significantly reduce unexpected hyperopia [28]. Accordingly, Diener et al. indicated Q value and R_PA_ parameter, which was calculated as the posterior to anterior corneal curvature radii ratio, as surrogate markers to identify eyes that might be at risk of a greater postoperative hyperopic shift after DMEK [29]. Bearing in mind the above studies, Campbell et al. evaluated the refractive accuracy of different IOL formulas and proposed Haigis formula to reduce the hyperopic error in patients undergoing the triple procedure [30].

Taken together, the observed inconsistences between studies might have been associated with the use of different topography devices, possible differences in operating technique and donor tissue preparation, different conditions of donor graft storage, or differences in the criteria for study group selection. Thus, we suggest that while carrying out such procedures, surgeons should create their own intraocular power nomograms for cataract surgery before or during DMEK surgery based on their definite observations and own experience.

Data on corneal aberrations after DMEK surgery differ between studies and are not consistent. In terms of astigmatism power astigmatism power, we documented a reduction in the total astigmatism power detected exclusively between the 1st and 3rd months after the procedure with subsequent stabilization of astigmatism magnitude. Contrary to our study, the recent report by Gundlach et al. presented the opposite pattern of a decrease in astigmatism from the 3rd to the 12th month postoperatively [31]. However, the authors scheduled only two postoperative examinations, while our study provided a more detailed analysis based on four follow-up time point observations. On the other hand, Guerra et al., in their prospective, consecutive, interventional series of 136 eyes, did not observe any significant changes between post- and preoperative astigmatism [11]. Accordingly, Shajari et al. concluded that the extent of corneal astigmatism change after the DMEK procedure in patients with Fuchs endothelial dystrophy is not predictable, which might explain the mentioned discrepancies between published data [20]. It is also worth mentioning that the axis of the baseline total astigmatism remained unchanged throughout the whole follow-up time in our study. Hence, it can be concluded that the DMEK procedure did not induce secondary astigmatism itself. Accordingly, when analyzing the asymmetry of astigmatism components, we found that astigmatism asymmetry remained stable 6 months postoperatively. With regard to higher-order aberrations, we documented that corneal HOA underwent a gradual reduction throughout the whole 12-month observation time, suggesting the ongoing process of tissue remodeling from a long-term perspective. This outcome corresponds with the gradational improvement in vision acuity recorded at subsequent visits in our study. It is noteworthy that previous analyses reporting the changes in HOA after the DMEK procedure are inconclusive. Contrary to our data, Duggan et al. reported no differences in HOA values documented at 12 months postoperatively compared to preoperative values for the total and anterior cornea. The only differences the authors found were for the posterior cornea 6 months after DMEK surgery. Likewise, Gundlach et al. [31] did not show differences between pre- and postoperative anterior and posterior HOA values at the 12-month follow-up. On the other hand, the investigation of Hayashil et al. documented a significant decrease in anterior, posterior, and total HOA recordings starting from the 3rd month after surgery. Accordingly, the authors found no changes in HOA values in early postoperative follow-up times up to the 3rd month [32]. The possible explanation for this discrepancy is diverse stages of endothelial decompensation at baseline in various studies strongly interrelated with differential preoperative visual acuity and corneal thickness. This possibility is supported by our observation that baseline CCT influences the changes in corneal topographic parameters after surgery and negatively correlates with the changes in corneal thickness, astigmatism power, astigmatism asymmetry, and HOA at subsequent postoperative visits. These observations are consistent with previous studies, as baseline CCT represents an efficient predictor for relevant outcome parameters after DMEK surgery [13]. Our findings support the view that different preoperative conditions of corneal oedema may result in different corneal curvature and pachymetry changes. Thus, the more “decompensated” corneas preoperatively are expected to present higher values of regular and irregular corneal astigmatism with an asymmetry of the astigmatic components and HOA after surgery.

Importantly, we detected that preoperative values of corneal topographic parameters strongly determined the changes detected after DMEK surgery since we found negative correlations between their baseline values and postoperative variations. This result strongly corroborates the notion that early therapeutic intervention results in better visual outcomes.

In conclusion, the results of our study provide valuable information regarding the dynamics of postoperative changes in corneal parameters after the DMEK procedure. The presented data clearly demonstrate that the stabilization of most corneal topographic parameters (i.e., mean keratometry, mean astigmatism, and asymmetry of astigmatism) takes place within 6 months after the procedure, whereas HOA and BCVA gradually improve during the first year after surgery.

## Figures and Tables

**Table 1:** Preoperative characteristics of the patient and donor corneas.

Parameter	Value
Preoperative BCVA (decimal)	0.22 ± 0.15
Preoperative CCT (*μ*m)	680.50 ± 89.6
Recipient age (y)	66.25 ± 11.23
Recipient sex (m/f)	5/16
Donor age (y)	60.08 ± 6.23
Donor sex (m/f)	11/13
Donor graft ECC (cells/mm^2^)	3057.83 ± 317.73
Type of cornea donation	19 multiple organ procurements, 5 procurements during the autopsy
Indications for the surgery (FECD/PBK)	19/4

BCVA = best corrected visual acuity (in decimal values); CCT = central corneal thickness; y = years; m = male; f = female; ECC = endothelial cell count; FECD = Fuchs endothelial corneal dystrophy; PBK = pseudophakic bullous keratopathy. Where possible, data are presented as the mean ± standard deviation (SD).

**Table 2:** The dynamics of changes in mean keratometry, astigmatism magnitude, astigmatism asymmetry, and higher-order aberration power between the follow-up time points in the different corneal optical zones after Descemet membrane endothelial keratoplasty.

			Baseline median (IQR)	1-month median (IQR)	3-month median (IQR)	6-month median (IQR)	12-month median (IQR)
Mean keratometry (D)	Anterior	3 mm OZ	49.96 (4.96)	48.34 (2.85)	48.11 (2.90)	49.02 (2.68)	49.26 (2.68)
		6 mm OZ	49.39 (3.60)	47.47 (2.33)	47.47 (2.64)	48.72 (2.19)	48.77 (2.48)
	Posterior	3 mm OZ	−6.06 (0.89)	**−6.47 (0.40)**	**−6.37 (0.35)**	**−6.32 (0.41)**	−6.34 (0.31)
		6 mm OZ	−6.01 (0.90)	**−6.51 (0.37)**	**−6.35 (0.40)**	**−6.29 (0.45)**	−6.32 (0.29)
	Total	3 mm OZ	43.88 (4.46)	42.07 (2.18)	41.92 (2.29)	42.76 (2.21)	42.92 (2.31)
		6 mm OZ	43.41 (3.38)	**41.17 (2.08)**	41.46 (2.18)	42.45 (1.93)	42.58 (2.28)

Mean astigmatism (D)	Anterior	3 mm OZ	1.47 (1.01)	1.58 (1.28)	**0.93 (0.83)**	0.92 (0.68)	0.88 (0.90)
		6 mm OZ	1.37 (0.97)	1.38 (1.25)	**0.87 (0.57)**	0.83 (0.84)	0.78 (0.81)
	Posterior	3 mm OZ	0.30 (0.30)	0.28 (0.20)	0.22 (0.13)	**0.19 (0.13)**	0.18 (0.14)
		6 mm OZ	0.26 (0.2)	0.25 (0.14)	**0.21 (0.115)**	**0.18 (0.09)**	0.17 (0.08)
	Total	3 mm OZ	1.31 (1.57)	1.63 (1.15)	**0.89 (0.825)**	0.93 (0.6)	0.835 (0.51)
		6 mm OZ	1.55 (1.08)	1.32 (1.08)	**0.83 (0.58)**	0.89 (0.65)	0.83 (0.755)

Astigmatism asymmetry (D)	Anterior	3 mm OZ	1.11 (1.79)	**1.00 (0.59)**	0.57 (0.44)	**0.50 (0.29)**	0.49 (0.30)
		6 mm OZ	1.50 (2.44)	**1.09 (0.57)**	**0.74 (0.63)**	0.74 (0.41)	0.63 (0.36)
	Posterior	3 mm OZ	0.43 (0.54)	**0.24 (0.23)**	**0.18 (0.09)**	0.15 (0.1)	0.13 (0.09)
		6 mm OZ	0.51 (0.66)	**0.30 (0.18)**	**0.22 (0.10)**	0.18 (0.1)	0.15 (0.11)
	Total	3 mm OZ	1.41 (1.69)	**0.78 (0.66)**	0.62 (0.42)	**0.51 (0.32)**	0.55 (0.39)
		6 mm OZ	1.92 (2.53)	**1.16 (0.76)**	**0.87 (0.60)**	0.74 (0.46)	0.67 (0.44)

Higher-order aberrations (D)	Anterior	3 mm OZ	0.48 (0.98)	0.34 (0.37)	0.28 (0.19)	0.28 (0.15)	**0.22 (0.10)**
		6 mm OZ	0.48 (0.7)	0.44 (0.56)	**0.38 (0.195)**	0.36 (0.22)	**0.31 (0.125)**
	Posterior	3 mm OZ	0.18 (0.16)	**0.09 (0.05)**	0.08 (0.06)	**0.08 (0.06)**	0.07 (0.05)
		6 mm OZ	0.17 (0.14)	**0.11 (0.06)**	**0.085 (0.05)**	0.09 (0.04)	0.09 (0.05)
	Total	3 mm OZ	0.46 (0.85)	**0.37 (0.33)**	**0.27 (0.15)**	0.27 (0.1)	**0.21 (0.08)**
		6 mm OZ	0.53 (0.81)	0.43 (0.55)	**0.37 (0.20)**	0.35 (0.18)	**0.305 (0.095)**

Statistically significant values are shown in bold. *p* values are calculated for intervals “baseline-1st month,” “1st month–3rd month,” “3rd month–6th month,” and “6th month–12th month.”

**Table 3:** The correlations between changes in VA, CCT, keratometry, astigmatism, astigmatism asymmetry, and higher-order aberration and baseline values of those parameters.

Correlation			The change of the selected parameter at 1 month as compared to baseline	The change of the selected parameter at 3 months as compared to baseline	The change of the selected parameter at 6 months as compared to baseline	The change of the selected parameter at 12 months as compared to baseline
Baseline BCVA			−0.11	−0.28	−0.19	−**0.57**
Baseline CCT			−**0.90**	−**0.87**	−**0.91**	−**0.91**
Baseline mean keratometry	Anterior	3 mm OZ	−**0.79**	−**0.84**	−**0.81**	−**0.85**
		6 mm OZ	**−0.72**	**−0.76**	**−0.75**	**−0.78**
	Posterior	3 mm OZ	**−0.86**	**−0.94**	**−0.93**	**−0.92**
		6 mm OZ	**−0.83**	**−0.87**	**−0.89**	**−0.89**
	Total	3 mm OZ	**−0.88**	**−0.89**	**−0.87**	**−0.90**
		6 mm OZ	**−0.86**	**−0.86**	**−0.87**	**−0.86**

Baseline mean astigmatism	Anterior	3 mm OZ	**−0.46**	**−0.84**	**−0.86**	**−0.70**
		6 mm OZ	−0.13	**−0.59**	**−0.65**	**−0.57**
	Posterior	3 mm OZ	**−0.64**	**−0.83**	**−0.83**	**−0.74**
		6 mm OZ	**−0.55**	**−0.83**	**−0.84**	**−0.75**
	Total	3 mm OZ	**−0.52**	**−0.86**	**−0.86**	**−0.78**
		6 mm OZ	−0.40	**−0.76**	**−0.81**	**−0.74**

Baseline astigmatism asymmetry	Anterior	3 mm OZ	−0.12	−0.14	−0.12	−0.16
		6 mm OZ	−0.01	−0.14	+0.05	+0.05
	Posterior	3 mm OZ	**−0.90**	**−0.95**	**−0.92**	**−0.94**
		6 mm OZ	**−0.92**	**−0.93**	**−0.91**	**−0.94**
	Total	3 mm OZ	**−0.91**	**−0.92**	**−0.95**	**−0.91**
		6 mm OZ	**−0.90**	**−0.94**	**−0.96**	**−0.91**

Baseline higher-order aberrations	Anterior	3 mm OZ	**−0.84**	**−0.87**	**−0.88**	**−0.97**
		6 mm OZ	**−0.80**	**−0.92**	**−0.92**	**−0.98**
	Posterior	3 mm OZ	**−0.89**	**−0.93**	**−0.90**	**−0.95**
		6 mm OZ	**−0.85**	**−0.87**	**−0.85**	**−0.89**
	Total	3 mm OZ	**−0.83**	**−0.85**	**−0.93**	**−0.98**
		6 mm OZ	**−0.81**	**−0.94**	**−0.95**	**−0.97**

The correlations were calculated for 4 consecutive time points, i.e., 1, 3, 6, and 12 months postoperatively. Significant values are shown in bold.

**Table 4:** The correlations between baseline CCT and the changes in VA, CCT, keratometry, astigmatism, astigmatism asymmetry, and higher-order aberration values obtained in the 3 and 6 mm optical zones after DMEK surgery.

Correlation of baseline CCT and			At baseline	At 1 month	At 3 months	At 6 months	At 12 months
BCVA change			−**0.54**	+0.20	+0.26	+0.20	+0.27
CCT change			1.00	−**0.90**	−**0.87**	−**0.91**	−**0.91**
Mean keratometry change	Anterior	3 mm OZ	+0.04	−0.005	−0.03	−0.01	−0.14
		6 mm OZ	−0.01	−0.07	−0.03	+0.01	−0.06
	Posterior	3 mm OZ	+0.04	−0.13	−0.07	−0.004	−0.05
		6 mm OZ	+0.08	−0.22	−0.12	−0.13	−0.18
	Total	3 mm OZ	+0.05	−0.07	−0.03	+0.02	−0.15
		6 mm OZ	+0.02	−0.09	−0.06	+0.002	−0.09

Mean astigmatism change	Anterior	3 mm OZ	**+0.42**	−0.18	**−0.45**	**−0.50**	**−0.56**
		6 mm OZ	**+0.48**	−0.30	**−0.52**	**−0.57**	**−0.63**
	Posterior	3 mm OZ	**+0.52**	**−0.62**	**−0.54**	**−0.52**	**−0.62**
		6 mm OZ	**+0.48**	**−0.48**	**−0.45**	**−0.44**	**−0.49**
	Total	3 mm OZ	**+0.49**	−0.31	**−0.54**	**−0.55**	**−0.65**
		6 mm OZ	**+0.66**	−0.41	**−0.62**	**−0.65**	**−0.70**

Astigmatism asymmetry change	Anterior	3 mm OZ	**+0.58**	−0.20	−0.29	+0.07	−0.11
		6 mm OZ	**+0.51**	−0.15	−0.31	+0.02	+0.02
	Posterior	3 mm OZ	**+0.58**	−0.36	**−0.51**	**−0.52**	**−0.58**
		6 mm OZ	**+0.62**	**−0.49**	**−0.52**	**−0.56**	**−0.66**
	Total	3 mm OZ	**+0.61**	**−0.58**	**−0.55**	**−0.53**	**−0.60**
		6 mm OZ	**+0.59**	**−0.45**	**−0.53**	**−0.54**	**−0.66**

Higher-order aberrations change	Anterior	3 mm OZ	**+0.44**	−0.33	**−0.44**	**−0.53**	**−0.54**
		6 mm OZ	**+0.48**	−0.31	−0.40	**−0.46**	**−0.54**
	Posterior	3 mm OZ	**+0.73**	**−0.64**	**−0.63**	**−0.57**	**−0.68**
		6 mm OZ	**+0.77**	**−0.77**	**−0.65**	**−0.58**	**−0.71**
	Total	3 mm OZ	**+0.56**	−0.35	**−0.48**	**−0.64**	**−0.61**
		6 mm OZ	**+0.57**	−0.36	**−0.48**	**−0.50**	**−0.60**

Correlations were calculated for 4 consecutive time points, i.e., 1, 3, 6, and 12 months postoperatively. At the zero observation time point, baseline CCT values refer to baseline absolute values of listed parameters. Significant values are shown in bold.

## Data Availability

The data used to support the findings of this study are available from the corresponding author upon request.
